# The spleen may be an important target of stem cell therapy for stroke

**DOI:** 10.1186/s12974-019-1400-0

**Published:** 2019-01-30

**Authors:** Zhe Wang, Da He, Ya-Yue Zeng, Li Zhu, Chao Yang, Yong-Juan Lu, Jie-Qiong Huang, Xiao-Yan Cheng, Xiang-Hong Huang, Xiao-Jun Tan

**Affiliations:** 1 Xiangtan Central Hospital, Clinical Practice Base of Central South University, Xiangtan, 411100 China; 2 Institute of Reproductive and Stem Cell Research, School of Basic Medical Science, Central South University, Changsha, 410000 China

**Keywords:** Stroke, Spleen, Stem cells, IL-10, Multipotent adult progenitor cells

## Abstract

Stroke is the most common cerebrovascular disease, the second leading cause of death behind heart disease and is a major cause of long-term disability worldwide. Currently, systemic immunomodulatory therapy based on intravenous cells is attracting attention. The immune response to acute stroke is a major factor in cerebral ischaemia (CI) pathobiology and outcomes. Over the past decade, the significant contribution of the spleen to ischaemic stroke has gained considerable attention in stroke research. The changes in the spleen after stroke are mainly reflected in morphology, immune cells and cytokines, and these changes are closely related to the stroke outcomes. Autonomic nervous system (ANS) activation, release of central nervous system (CNS) antigens and chemokine/chemokine receptor interactions have been documented to be essential for efficient brain-spleen cross-talk after stroke. In various experimental models, human umbilical cord blood cells (hUCBs), haematopoietic stem cells (HSCs), bone marrow stem cells (BMSCs), human amnion epithelial cells (hAECs), neural stem cells (NSCs) and multipotent adult progenitor cells (MAPCs) have been shown to reduce the neurological damage caused by stroke. The different effects of these cell types on the interleukin (IL)-10, interferon (IFN), and cholinergic anti-inflammatory pathways in the spleen after stroke may promote the development of new cell therapy targets and strategies. The spleen will become a potential target of various stem cell therapies for stroke represented by MAPC treatment.

## Introduction

Stroke is the most common cerebrovascular disease and the second leading cause of death behind heart disease and is a major cause of long-term disability worldwide [1]. Our understanding of the pathophysiological cascade following ischaemic injury to the brain has greatly improved over the past few decades. Cell therapy, as a new strategy addition to traditional surgery and thrombolytic therapy, has attracted increasing attention [2]. The therapeutic options for stroke are limited, especially after the acute phase. Cell therapies offer a wider therapeutic time window, may be available for a larger number of patients and allow combinations with other rehabilitative strategies.

The immune response to acute stroke is a major factor in cerebral ischaemia (CI) pathobiology and outcomes [3]. In addition to the significant increase in inflammatory levels in the brain lesion area, the immune status of other peripheral immune organs (PIOs, such as the bone marrow, thymus, cervical lymph nodes, intestine and spleen) also change to varying degrees following CI, especially in the spleen [4]. Over the past decade, the significant contribution of the spleen to ischaemic stroke has gained considerable attention in stroke research. At present, the spleen is becoming a potential target in the field of stroke therapy for various stem cell treatments represented by multipotent adult progenitor cells (MAPCs).

### Two cell therapy strategies

**Fig. 1:**
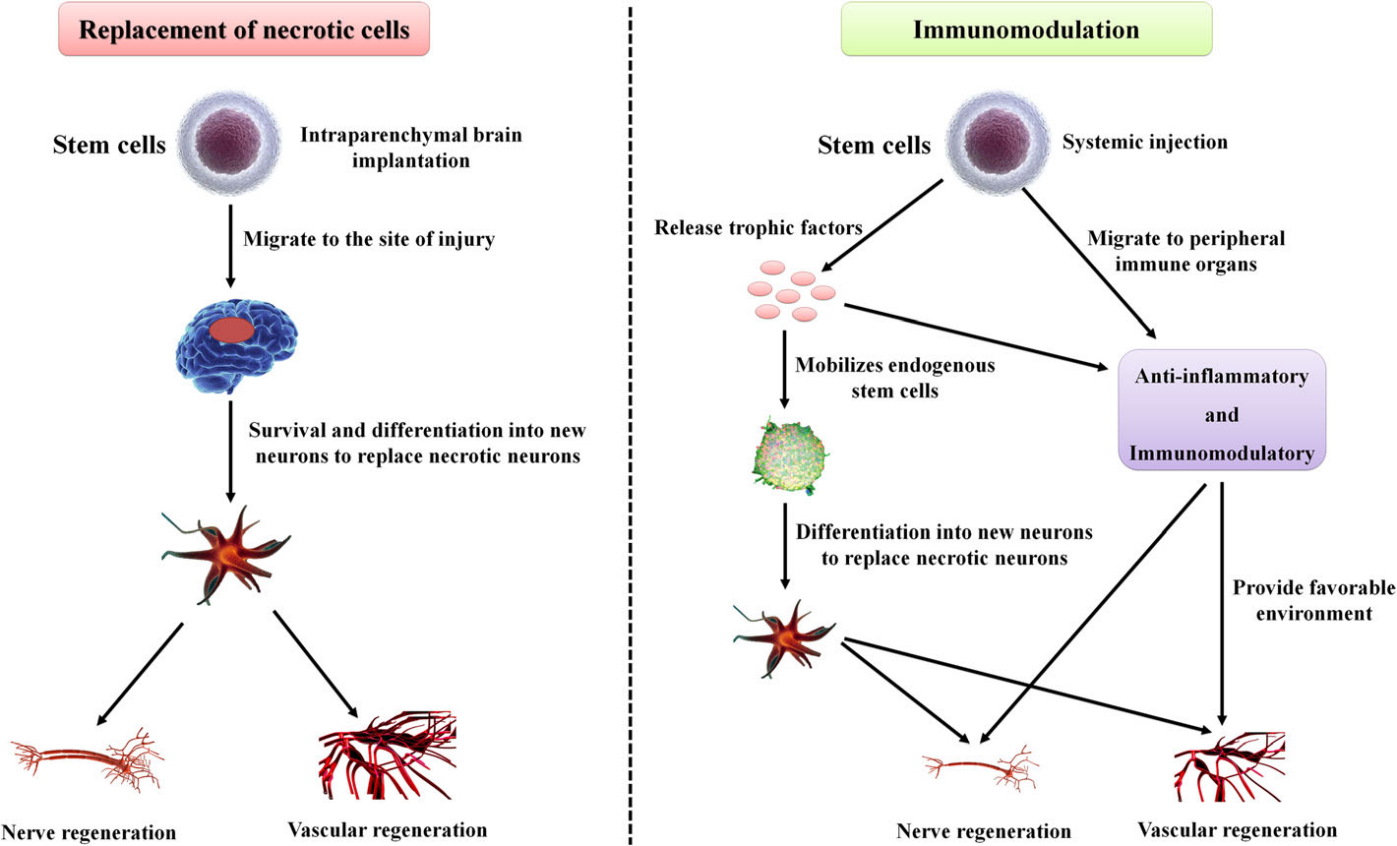
Two cell therapeutic strategies for stroke. Replacement of necrotic cells and immunomodulation. Therapeutic stem cells have traditionally been known to differentiate into cells that make up nerve tissue to replace necrotic cells, thereby promoting nerve regeneration and angiogenesis. Recent studies have shown that the immune regulatory capacity of stem cells provides a favourable environment for nerve and vascular regeneration

**Fig. 2:**
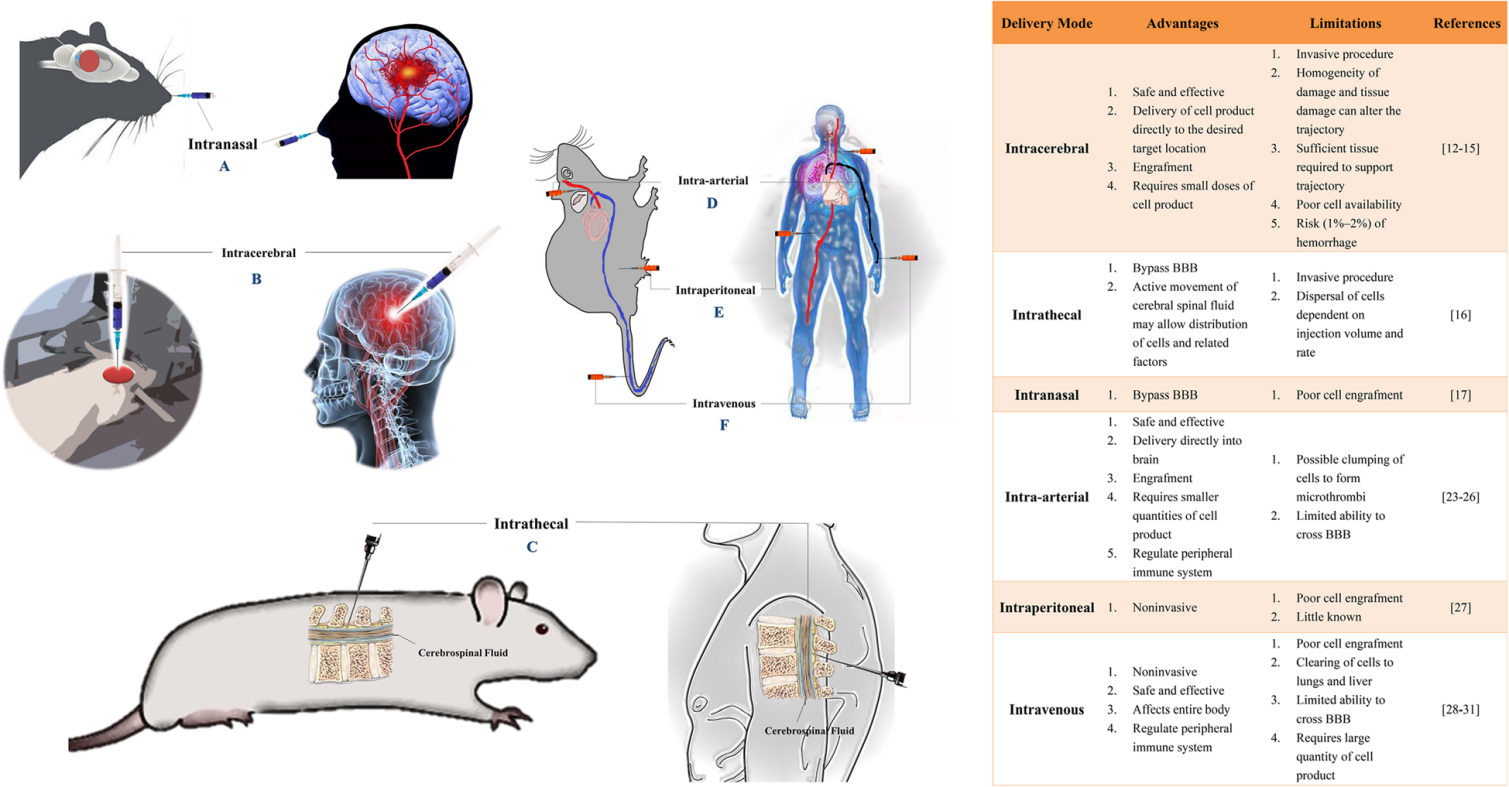
The main routes of administration of stem cell therapy for stroke. Although many preclinical studies and clinical applications have been carried out, the most adequate administration route for stroke is unclear. Each administration route has advantages and disadvantages for clinical translation to stroke patients. **a** Intranasal, **b** intracerebral, **c** intrathecal, **d** intra-arterial, **e** intraperitoneal and **f** intravenous

Two distinct cell therapy strategies have emerged from clinical data and animal experiments (Fig. 1). The first is the nerve repair strategy, which uses different types of stem cells with the ability to differentiate into cells that make up nerve tissue and thus can replace damaged nerves to promote recovery during the later stages after stroke [5–11]. This strategy usually involves cell delivery to the injury site by intraparenchymal brain implantation and stereotaxic injection into unaffected deep brain structures adjacent to the injury site. The main problem with this strategy is that we should not only ensure the efficient delivery of cells to the injury site but also try to reduce the invasive damage caused by the mode of delivery. Moreover, evaluation of the extent to which cells survive over the long term, the differentiation fates of the surviving cells and whether survival results in functional engraftment is difficult. This strategy mainly includes intracerebral [12–15], intrathecal [16] and intranasal administration [17] (Fig. 2).

The second strategy is an immunoregulatory strategy (typically therapeutic cells are injected intravenously), which takes advantage of the release of trophic factors to promote endogenous stem cell (NSC/neural progenitor cell (NPC)) mobilisation and anti-apoptotic effects in addition to the anti-inflammatory and immunomodulatory effects encountered after systemic cell delivery. The mechanism of action appears to be reliant on “bystander” effects; these effects are likely to include immunomodulatory and anti-inflammatory effects mediated by the systemic release of trophic factors [18, 19], since neither animal nor human data have found any signs of actual engraftment of intravenously delivered cells in the brain [20–22]. In addition, many therapeutic stem cells have been found to migrate to PIOs, such as the spleen, following brain injury to play an immunoregulatory role, thus providing a good environment for nerve and vascular regeneration in vivo. This strategy mainly includes intra-arterial [23–26], intraperitoneal [27] and intravenous administration [28–31] (Fig. 2). Currently, systemic immunomodulatory therapy based on intravenous cells is attracting increasing attention [29].

### Immunoregulation may be a better strategy

Further insight into the role of the two strategies has been provided by studies using cellular therapies in experimental models of brain ischaemia. All cells are more efficacious when administered systemically than when delivered via intracerebral administration [32–37], probably because intracerebral administration does not guarantee the extent to which cells can migrate from their implantation site in human subjects. Placing cells within the cystic space left as a long-term consequence of ischaemic damage in the absence of some type of bio-scaffold will be unlikely to promote cell adherence or persistence. Moreover, gliosis on the margins of the damaged region may impede cell migration or axonal outgrowth in the same manner as encountered after spinal cord injury.

The pathological progression of stroke is a complex systemic process, and changes in state occur in tissues besides intracranial tissues. Studies have shown that the immune response/regulation after stroke plays an important role in the pathological progression of stroke. The immune response is an important endogenous mechanism of post-stroke activation. Although the immune response following stroke, including cytokine production and inflammatory cell infiltration into damaged brain tissues, has been known for many years [38–40], the complexity of the mechanisms involved in post-stroke immune activation, inflammatory damage and tissue repair are unknown. In the future, immunomodulation will be an important potential therapeutic strategy for stroke. Moreover, finding the most appropriate therapeutic target for therapeutic cells may further improve the effectiveness of immunomodulatory treatment.

### Stem cells and immunoregulation after stroke

At present, most cells used for immunoregulation therapy after stroke are various types of stem cells. However, animal experiments have shown that anti-inflammatory immune cells (such as regulatory T cells (Tregs), helper T (Th)-2 cells and regulatory B cells (Bregs)) can also alleviate brain damage [41–44]. In addition, some immune cells are activated after stroke, such as monocytes in some PIOs or astrocytes and have been shown to have protective effects in experimental animals [45–47].

Stem cell therapy has received considerable attention and application because of the easy access, strong proliferation and low immunogenicity of the cells. Treatments based on different types of stem cells have been studied for years and even decades in animal models of stroke. Included in the following subsections are specific examples of cell therapies that have been extensively studied in animal models and taken forward to clinical trials. For instance, human umbilical cord blood cells (hUCBs) [32–34], haematopoietic stem cells (HSCs) [35], bone marrow stem cells (BMSCs) [36], human amnion epithelial cells (hAECs) [48] and neural stem cells (NSCs) [37] have all been shown to reduce neural injury in experimental models of stroke.

Interestingly, almost all studies have found that when administered systemically, stem cells migrate to the injured brain and PIOs and in some cases have been shown to modulate the immune response to stroke [32, 35–37, 48], which may be one reason that this injection route is more efficacious. Studies have also shown that only a small number of stem cells injected intravenously after a stroke can be transported through the blood-brain barrier to damaged brain tissue [31]. This finding suggests that regulation of the peripheral immune status after stroke may be a potentially important therapeutic strategy, especially for improvement of the long-term prognosis in stroke patients.

In addition to stem cells themselves, exosomes derived from some stem cells have been found to have therapeutic effects on haemorrhagic stroke [49]. For instance, transplantation of pluripotent mesenchymal stem cell (MSC)-derived exosomes promoted functional recovery in an experimental intracerebral haemorrhage (ICH) rat model [50]. MSC-derived exosomes can amplify endogenous brain repair mechanisms and induce neurorestorative effects after CI [51]. Exosomes carry a concentrated group of functional molecules (DNA, ribosomal RNA, circular RNA, long noncoding RNA, microRNA, proteins and lipids) that serve as intercellular communicators not only locally but also systemically. These molecules may be part of the long-distance cell-to-cell communication that operates by paracrine function through secretory factors in the extracellular environment and is responsible for the long-distance effects during cell therapy.

### Stroke and inflammation

**Fig. 3:**
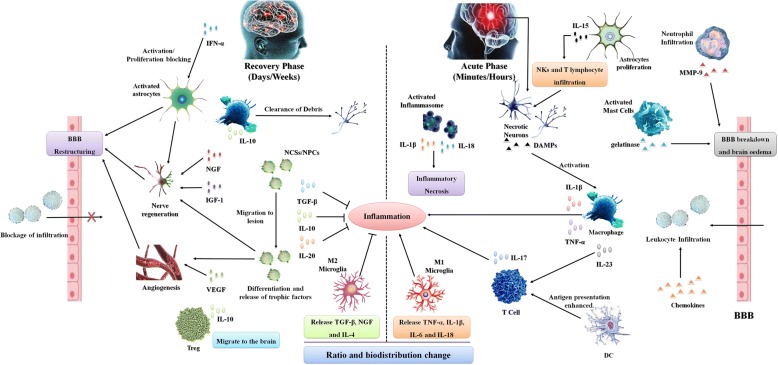
Inflammation after stroke. DAMPs released from necrotic neurons activate macrophages through PRRs and the inflammasome. Activated macrophages enhance inflammation by releasing pro-inflammatory cytokines and recruiting T cells, which contribute to maintenance of inflammation through IL-17. DCs also activate and enhance antigen presentation to T cells. Gelatinase released by activated mast cells and MPP-9 produced by infiltrating neutrophils destroy the function of the BBB, resulting in brain oedema. Then, under the action of chemokines, leukocytes infiltrate into the damaged brain tissue, thereby expanding inflammation and injury. Several days after acute stroke, the cytokines produced by the innate immune system change to an anti-inflammatory phenotype, which contributes to inhibition of inflammation. The ratio and biodistribution of M1 and M2 microglia also changes, with anti-inflammatory M2 microglia becoming dominant again. Debris is cleaned up by microglia and macrophages. NSCs/NPCs are mobilised and migrate to the lesion. The environment becomes conducive to nerve regeneration, angiogenesis and BBB restructuring

The pathophysiological process of stroke is very complex and involves energy metabolism disorders, acidosis, loss of cellular homeostasis, excitotoxicity, activation of neurons and glial cells, blood-brain barrier (BBB) destruction and accompanying leukocyte infiltration [52]. Evidence suggests that the immune system is involved in the various pathological stages of stroke [53]. CI initiates an inhibitory effect on lymphatic organs through the autonomic nervous system (ANS), which increases the risk of infection after stroke. Infection after stroke is a major cause of disability and death after stroke [54]. On the other hand, the innate immune system also contributes to repair of brain tissue [55] (Fig. 3).

#### Inflammatory cell infiltration and tissue damage

The inflammatory response to stroke starts immediately in the lacuna after arterial occlusion, and production of reactive oxygen species (ROS) increases rapidly in the coagulation-promoting state, accompanied by activation of complement, platelets and endothelial cells [56, 57]. Increased cyclooxygenase-2 (COX-2) activity in inflammatory cells and neurons may lead to increased ROS production in the injured tissues and severe prostaglandin toxicity [58, 59]. ROS also help reduce nitric oxide (NO) activity, leading to platelet aggregation and leukocyte adhesion and thus aggravation of ischaemic injury [60]. After a few minutes of arterial occlusion, the relevant intracellular and extracellular regulation begins immediately. Acute local injury is sensed by pattern recognition receptors (PRRs) by interaction with pathogen-associated molecular patterns (PAMPs) and damage-associated molecular patterns (DAMPs) [61–63]. These factors are released by stressed cells in the blood cascade, and PRRs in neurons and glial cells can activate intracellular signal transduction pathways to increase the expression of different pro-inflammatory genes [64, 65]. This mechanism activates immune system factors that cause mast cells to release vasoactive mediators, such as histamine, protease and tumour necrosis factor (TNF), whereas macrophages release pro-inflammatory factors [66]. After the rapid production of inflammatory signals, the interaction between adhesion molecules and integrins is mediated by adhesion receptors to facilitate leukocyte infiltration into the brain parenchyma [67, 68]. After ischaemia, these cells rapidly release pro-inflammatory mediators into the area, and these cytokines contribute to leukocyte infiltration into damaged tissues and activate antigen presentation between dendritic cells (DCs) and T cells [69, 70]. T cells cause tissue damage through IFN-γ and ROS. IL-23 released by microglia and macrophages activates T cells to produce IL-17, which aggravates the acute ischaemic brain injury [71]. Ultimately, this neuroimmune imbalance leads to an early downregulation of systemic cellular immune responses, resulting in functional deactivation of monocytes, Helper T (Th) cells and invariable natural killer T cells (iNKTs) [72]. This stage is often accompanied by increased lymphocyte apoptosis, inhibition of peripheral cytokine release and helper Th1 cells and changes in the Th1/Th2 ratio. Stroke-induced immunosuppression helps to increase the risk of infection, leading to adverse functional outcomes [73].

#### Phenotypic and spatial distributions of microglia

Microglia can be regarded as resident immune cells in the central nervous system (CNS) that are activated by local and systemic infections, neurodegenerative diseases and tissue damage. Microglia respond quickly to stroke injury. Microglia enter the ischaemic centre within 60 min after focal ischaemia, and the number of activated microglia increases significantly for up to 24 h. Pro-inflammatory M1 microglia (which release TNF-α, IL-1β, IL-6 and IL-18) [74] can be observed in the ischaemic core within 24 h after CI, and the number of M1 microglia increases gradually within 2 weeks of CI [75]. Inhibitory M2 microglia (which participate in neuroprotection and promote repair of damaged cells through production of transforming growth factor (TGF)-β, nerve growth factor (NGF) and IL-4) [74] begin to appear 24 h after injury, and their number gradually increases over time for up to 1 week after ischaemia [38]. The phenotypes and spatial distributions of microglia change with the expansion of damaged brain tissue [76, 77].

#### Astrocytic proliferation and activation

Astrocytes are the most abundant cell type in the CNS and perform multiple functions that are both detrimental and beneficial for neuronal survival from the acute phase to the recovery phase after ischaemic stroke [78]. IL-15 expression is increased in astrocytes in mouse and human brains after CI, which elevates the level and activation of CD8^+^ T cells and natural killer cells (NKs), resulting in aggravation of brain tissue damage [79, 80]. IL-15 blockade reduces the effects of NKs, CD8^+^ T cells and CD4^+^ T cells in the brains of mice after ischaemia/reperfusion (I/R), resulting in a reduction of the infarct size and improvement in motor and locomotor activity [80]. During the recovery phase, IFN-α is mainly involved in regulation of astrocytic proliferation through blocking and activation [29]. Astrocytes regulate the formation and maintenance of synapses, cerebral blood flow and BBB integrity [81]. Astrocytes also indirectly regulate inflammation by affecting neuronal survival during acute injury and axonal regrowth [81]. Activated astrocytes are beneficial for the recovery of neurological function after stroke [82]. Recent studies have suggested that this endogenous protective mechanism may involve mitochondrial transport from astrocytes to neurons after brain injury, which is mediated by a calcium-dependent mechanism involving CD38 and cyclic ADP ribose signalling [83].

#### Mast cells and BBB breakdown

Mast cells, which are located in the perivascular space surrounding the brain parenchymal vessels and in the dura mater of the meninges, are activated during the early stage after stroke and contribute to BBB breakdown and brain oedema by releasing gelatinase [84, 85]. Pharmacological mast cell stabilisation with cromoglycate reduces haemorrhage formation and mortality after administration of thrombolytics in experimental ischaemic stroke [86], which may involve promotion of BBB breakdown and neutrophil infiltration by mast cells [87].

#### Inflammasome activation

Inflammatory reactions lead to the production of inflammatory cytokines and the death of neurons and glial cells, which are regulated by a multiprotein complex called the inflammasome [67]. Nod-like receptors (NLR) in neurons and glial cells may mediate production of the inflammasome, which participates in the inflammatory response to aseptic tissue damage during CI [64]. The inflammasome in damaged brain tissue produces IL-1β and IL-18 after activation, which can cause specific cell death called inflammatory necrosis [88]. In this way, the inflammasome not only helps activate and support innate immunity but also aggravates tissue damage.

#### Inflammation relief and tissue repair

Inflammation after stroke is also inhibited by auto-suppression, and its remission is regulated by many immunosuppressive factors. The termination of inflammation also triggers structural and functional remodelling of damaged brain tissue. The first mechanism involved in this stage is the clearance of dead cells and is accomplished by microglia and infiltrating macrophages, which are mainly composed of phagocytes [76, 89]. Immunoglobulins targeting CNS antigens may promote the release of IL-10 and TGF-β, thereby inhibiting the immune response and the production of adhesion molecules and inflammatory cytokines [90]. These multipotent immunoregulatory factors can inhibit inflammation and contribute to tissue repair, and their protective effects are conducive to cell survival in ischaemic areas [60]. These growth factors are released by inflammatory cells, neurons and astrocytes and support cell budding, neuronal growth, angiogenesis and even tissue remodelling after ischaemic injury [91]. Insulin-like growth factor (IGF)-1 plays a key role in the neurogenesis process after ischaemic injury, and the astrocyte response is also necessary for the functional recovery of damaged tissues [92]. The roles of vascular endothelial growth factor (VEGF) and neutrophil metalloproteinase are also required for angiogenesis; together, they support the joint activity of inflammatory cells and astrocytes [93].

### Changes in peripheral immune organs after stroke

**Fig. 4:**
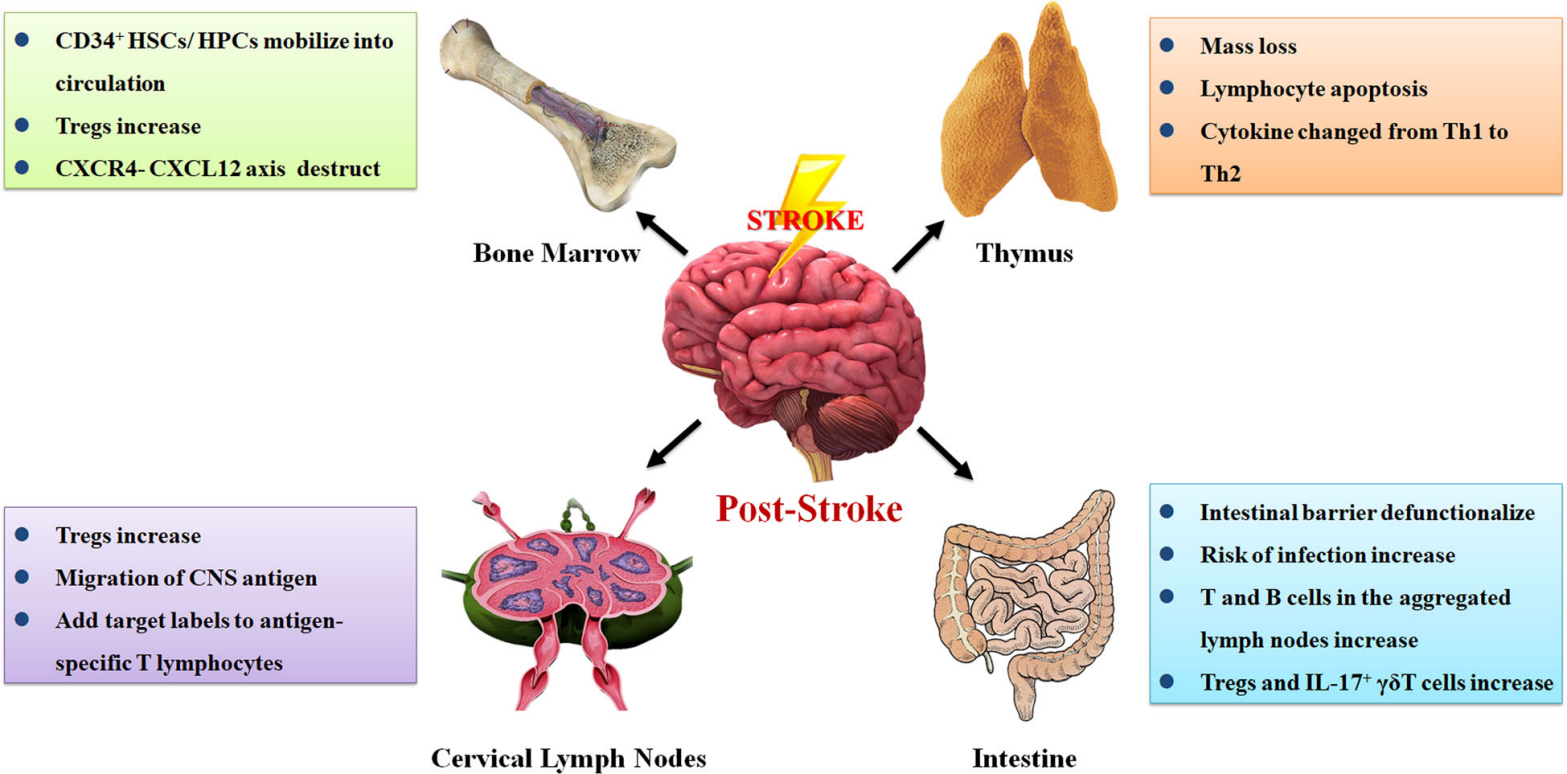
Changes in PIOs after stroke. Morphological and biochemical changes occur in the bone marrow, thymus, cervical lymph nodes and intestine after stroke and play respective roles in the stroke outcome

The pathological process after stroke is a complex systemic immune state change. In addition to severe inflammation in brain tissues (including inflammatory chemokine production, inflammatory cell infiltration, microglial activation and inflammasome production) [94], the state of PIOs also changes significantly after stroke [95] (Fig. 4).

#### Bone marrow

CD34^+^ HSCs/ haematopoietic progenitor cells (HPCs) in bone marrow are mobilised rapidly into the peripheral blood circulation under post-stroke pathological stress and play an important protective role in the pathological process of CI [96]. The prognosis can be effectively improved by accelerating the mobilisation of protective cells in bone marrow or increasing their levels in peripheral circulation after stroke [97, 98]. Clinical trials have also shown that intra-arterial injection of bone marrow-derived CD34^+^ haematopoietic stem cells/progenitor cells can significantly improve the prognosis of acute ischaemic stroke patients and greatly reduce their mortality and disability rates [26]. In addition, CI regulates the elevation of CD4^+^CD25^+^FoxP3^+^ Tregs from bone marrow via the sympathetic nervous system (SNS) [95]. Stroke reduces C-X-C chemokine ligand (CXCL) 12 expression in bone marrow but increases C-X-C chemokine receptor (CXCR) 4 expression in Tregs and other bone marrow cells. Destruction of the CXCR4-CXCL12 axis in bone marrow promotes mobilisation of Tregs and other CXCR4^+^ cells into peripheral circulation and eventually migration to damaged brain tissues to facilitate tissue repair [95].

#### Thymus

Animal data have shown that the thymus exhibits loss of a large number of lymphocytes within 12 h after ischaemia/reperfusion (I/R). Cytokine production also changes from the Th1 to the Th2 phenotype [99, 100]. Lymphocytes, such as B cells, T cells and natural killer cells (NKs), were found to be highly apoptotic [101], and the thymic morphology of the tested mice exhibited significant atrophy after I/R [102]. The non-toxic apoptosis inhibitor Q-VD-OPH significantly reduced programmed death of thymocytes after I/R, effectively reducing the incidence of bacteraemia after CI injury and improving the survival rate of the mice [103].

#### Cervical lymph nodes

Treg levels in brain tissue and cervical lymph nodes increase significantly after CI [104]. This increase may be due to changes in BBB permeability after stroke as well as other pathological causes, resulting in a large number of efflux cells and soluble proteins migrating from the brain tissue to the cervical lymph nodes. These cells and proteins migrate to the cervical lymph nodes and play an important role in regulating the pathological immune response after stroke [105, 106]. Many brain-derived antigens that migrate to the cervical lymph nodes after stroke may promote autoimmunity and Treg-based immunomodulation [107]. In addition, antigen-specific T lymphocytes may circulate from other parts of the body to the cervical lymph nodes, where they enter targeted cells via integrin expression on their surfaces and are transported to the damaged hemisphere [108].

#### Intestine

Experimental and clinical evidence has shown that temporary impairment of the immune response is an important factor in the high post-stroke infection rate [53, 109]. The intestine is often exposed to a large number of microorganisms and thus provides potential access to pathogens. Therefore, intestinal barrier dysfunction may be an important risk factor for bacterial translocation and endogenous infection. The numbers of T and B cells in aggregated lymph nodes have been shown to decrease significantly after CI, whereas the numbers of NKs and macrophages do not differ significantly. Compared with that of the control group, no significant change occurred in the lymphocyte subsets of the intestinal epithelium and lamina propria in rats with CI [110]. Stroke may have different effects on the immune cell composition in the intestinal lymphoid tissue, and this change may increase the susceptibility to infection after stroke [110].

In addition to the immune cell structure, the intestinal flora also plays an important role in the stroke prognosis. The interaction between the immune system and intestinal epithelial surface symbiotic microorganisms is essential for the development, maintenance and functionalization of immune cells [111, 112]. Intestinal symbiotic microorganisms are the most abundant symbiotic chambers in the human body and have potential as a method to regulate the levels of lymphocytes, including Tregs and γδT cells, which play key roles in the pathological process of stroke [67]. Altering the intestinal symbiotic microbial structure of mice using amoxicillin-clavulanic acid compound antibiotics induces tolerance and protection of the mice against I/R injury [112]. This protective effect can be transferred directly between mice through faecal feeding behaviour. Other antibiotics, such as vancomycin, can play a similar role in altering the structure of the intestinal flora and inducing tolerance to I/R in mice [112]. This protective mechanism may be due to alteration of the intestinal symbiotic microflora structure, resulting in the production of Tregs in intestinal lymph nodes derived from the small intestine. Treg homing in the intestine inhibits the differentiation of IL-17^+^ γδT cells via IL-10 secretion. After stroke, effector T cells migrate from the intestine to the meninges, because the decrease in IL-17^+^ γδT cells reduces CXCL1 and CXCL2 expression in ischaemic brain tissue, thereby reducing the migration and infiltration of leukocytes into the ischaemic brain tissue and the resulting brain tissue damage [112].

### The role of the spleen in stroke

Splenectomy has been shown to play a protective role in various brain injury models, including permanent/temporary middle cerebral artery occlusion (p/t MCAO), ICH and traumatic brain injury (TBI) [113–118]. Splenectomy before pMCAO significantly reduces the infarct size, numbers of neutrophils and activated microglia in the damaged brain tissue [113], IFN-γ level and number of infiltrating immune cells [119]. Splenectomy before tMCAO results in a significantly lower cerebral infarction volume and IFN-γ level after ischaemia and does not increase the risk of post-stroke infection [114]. Splenectomy immediately after different TBI injury models can also reduce nerve injury. For instance, vascular injury and brain oedema in the cerebral ischaemic region were significantly reduced in the splenectomy group [116–118]. Similar protective effects were observed in aged rats either before tMCAO or immediately after reperfusion with splenectomy [120]. However, splenectomy fails to provide long-term protection against I/R. In one study, splenectomy was performed 3 days after reperfusion, and the infarct volume, nerve function and peripheral blood immune cell count were assessed 28 days after stroke. The results showed that delayed splenectomy neither reduced brain tissue loss nor alleviated sensorimotor and cognitive impairment [121]. Although splenectomy immediately upon reperfusion significantly reduced the infarct size and immune cell infiltration 3 days after MCAO, the procedure failed to promote long-term recovery [121]. This finding indicates that the acute neuroprotective effect achieved by immediate splenectomy after stroke does not provide long-term protection and that immune regulation by the spleen may play different roles in different pathological stages of stroke. As an alternative to splenectomy, exposure of the spleen to radiation 4 h after tMCAO has similar protective effects. Exposure of the spleen to radiation causes a temporary decrease in splenic cells and does not cause extensive immunosuppression [122].

The changes in the spleen after stroke are mainly reflected in three aspects: first, the splenic morphology; second, the numbers of immune cells derived from the spleen; and third, inflammatory cytokine production by the spleen.

#### Splenic morphology

In different stroke animal models, splenic atrophy similar to that of the thymus appears after brain injury [102, 114]. The splenic morphology decreases gradually 24 to 72 h after pMCAO. The increase in catecholamines in the insular cortex after I/R may be an important cause of splenic atrophy after injury. Activation of alpha 1 adrenergic receptor (α1-AR) in the splenic smooth muscle sac causes contraction of the splenic envelope, which leads to a reduction of the splenic volume. Prazosin, which is an α1-AR antagonist, can effectively alleviate splenic atrophy after CI [123]. Clinical studies have also assessed changes in the shape of the spleen in stroke patients. One study showed that loss of splenic volume in ischaemic stroke patients began less than 6 h after stroke and that the process of splenic atrophy continued until approximately the third day after stroke, gradually increased from the fourth day to the eighth day and then basically returned to the pre-stroke state. At the same time, the size of the spleen after tMCAO is negatively correlated with the infarct volume, and more severe atrophy of the spleen is associated with a larger infarct volume [32]. The spleen of patients with ischaemic stroke may initially contract after onset and then re-expand, which contributes to ischaemic brain damage via splenic cell components [124]. Atrophy of the spleen is also accompanied by apoptosis of splenic cells and loss of B cells in the germinal centre. Moreover, this study also showed that the only subset of immune cells that decreased after tMCAO was B cells [102].

#### Immune cells

The spleen is the largest natural reservoir of immune cells, many of which also change in the spleen after stroke, including lymphocytes, monocytes, neutrophils and NKs. These cells are mobilised from the spleen to the brain after CI and play an important role in the pathological process after stroke [125]. Removal of blood neutrophils with an anti-neutrophil antibody has been shown to alleviate nerve damage and splenic atrophy in hypoxic ischaemic neonatal rats [126]. Neutrophils are the first immune cells to respond to ischaemic injury and infiltrate into the damaged brain within hours of stroke. Animal models show that neutrophil infiltration reaches a peak during days 1–3 after CI. In acute ischaemic stroke patients, neutrophils are recruited within 24 h after symptom onset [127]. Studies have shown that neutrophil infiltration plays an important role in the pathological process of stroke [113, 127]. Clinical data show that the splenic volume is negatively correlated with the percentage of blood lymphocytes and positively correlated with the percentage of neutrophils after acute stroke [128]. In addition, neutrophils may increase BBB permeability by releasing matrix metalloproteinase (MMP)-9, resulting in more leukocyte infiltration and increased neuroinflammation [129, 130]. Animal data also show that arginase I released by activated neutrophils after CI can induce peripheral immunosuppression [131]. As expected, the protective effect of splenectomy 2 weeks before pMCAO is also reflected in a reduction of neutrophils at the injury site [113].

Peripheral blood monocytes/macrophages have also been shown to migrate to and infiltrate into ischaemic brain tissue under the action of C-C chemokine ligand 2 (CCL-2) and to promote inflammation and tissue damage in the brain after stroke [132]. Researchers have promoted tissue repair and remodelling after brain ischaemia by injecting clodronate liposomes into mice to deplete macrophages and especially by reducing the numbers of macrophages in the spleen [133]. Interestingly, both pro-inflammatory Ly-6C^hi^ and anti-inflammatory Ly-6C^low^ were mobilised to migrate from the spleen to ischaemic brain tissue. However, another study reported that complete removal of splenic-derived monocytes/macrophages by splenectomy did not provide any protection against ischaemic brain tissue [45]. Therefore, only selective clearance of pro-inflammatory Ly-6C^hi^ and anti-inflammatory Ly-6C^low^ monocytes/macrophages can determine the different effects of different groups of cells on the stroke prognosis. However, systemic injection of low-dose lipopolysaccharide (LPS) induces a Ly6C^hi^ monocyte response that protects the brain after tMCAO in mice [134]. Remarkably, adoptive transfer of monocytes isolated from LPS-preconditioned mice into naïve mice 7 h after tMCAO reduces brain injury, although the protective effect still depends on an intact spleen [134].

Many studies have confirmed that T lymphocytes play a harmful role in I/R damage [135–137]. T cells contribute to the lymphopaenia induced by CI and are the most crucial lymphocytes for immunodepression after stroke [135]. In young mice, RTL1000 (a type of recombinant T cell receptor ligand) therapy inhibited the splenocyte efflux while reducing the frequency of T cells and macrophages in the spleen. Older mice treated with RTL1000 exhibited a significant reduction in inflammatory cells in the brain and inhibition of splenic atrophy [138]. The protective effect of splenectomy on the brain is also accompanied by a decrease in the number of T cells at the brain injury site [139]. However, some studies have also shown that certain T cell subsets, such as Tregs, may play a protective role in the pathological process of stroke [140, 141]. Animal data showed that the therapeutic effect of adoptive injection of Tregs could be maintained for at least 12 days [41]. The increase in the number of Tregs in the spleen after stroke is known and may reflect an endogenous protective mechanism [102]. Intraperitoneal injection of CD28SA (a CD28 superagonistic monoclonal antibody) after MCAO in mice increased the Treg levels in the brain and spleen, thereby attenuating the inflammatory response and improving the outcome after experimental stroke [142]. Gu et al. studied the effects of the absence of T cell subsets on brain infarction after in vivo stroke and then used an in vitro coculture system of splenocytes and neurons to further identify the roles of T cell subsets in neuronal death. The data displayed the detrimental versus beneficial effects of Th1 and Th2 cells both in vivo and in vitro [143].

B cells are the main type of splenic lymphocyte, but few studies have focused on the role of B cells in stroke injury. The lack of B cells does not improve brain injury in mice after I/R, suggesting that endogenous B cells do not have harmful effects after acute CI [145]. However, the animal data showed a genetic deficiency, and pharmacologic ablation of B lymphocytes using an anti-CD20 antibody prevented the appearance of delayed cognitive deficits [144]. Similar to Tregs, Bregs that secrete IL-10 also protect against I/R injury [145], but further studies are needed to confirm whether these Bregs are released by the spleen after stroke. However, exogenous transplantation of spleen-derived Bregs via intravenous injection has been shown to protect against CI [42, 146]. In addition, adoptive injection of Bregs into tMCAO rats can increase the level of CD8^+^CD122^+^ Tregs in the spleen and CNS after CI [147].

NKs, which also travel from the spleen into the ischaemic brain, are a type of cytotoxic cell that forms part of the innate immune system. Studies have shown that chemokines produced by ischaemic neurons cause NKs to migrate and infiltrate into the brain, where they promote further brain damage [148, 149]. In addition, splenic T lymphocytes are known to be activated by antigen-presenting cells (APCs), especially DCs. An increase in the number of DCs has been observed in both pMCAO and tMCAO [150]. Immature DCs patrol the blood and invade injured tissues, where they pick up antigens and acquire the ability to stimulate T cells in lymphoid tissues, such as the lymph nodes and spleen [151, 152]. Therefore, DCs can present antigens to T lymphocytes in the spleen and activate adaptive immunity after stroke. However, the exact role of splenic DCs in the prognosis of stroke is unknown.

From these studies, we can deduce that brain-spleen cell cycling after stroke can affect systemic inflammation and the brain inflammatory milieu, which may be a target for a novel therapeutic strategy.

#### Cytokines

Immune cells in the spleen contribute to the rise of cytokines in the blood and in turn in the brain after stroke. For example, spleen cells collected from stroke model mice show a stronger ability to secrete inflammatory cytokines, including TNF-α, IL-6, monocyte chemoattractant protein-1 (MCP-1) and IFN-γ [153], than those collected from normal mice. Many of these inflammatory cytokines and chemokines, such as IFN-γ and IFN-induced protein 10 (IP-10), have been shown to be key factors in stroke-induced neurodegenerative diseases [119, 153, 154]. Offner et al. also confirmed that the IL-2 and IL-10 levels in the spleen of experimental animals increased to varying degrees after CI [155]. In a clinical trial involving 158 healthy volunteers and 158 stroke patients, the levels of various inflammatory factors were elevated in patients with splenic contraction, with significant differences in IFN-γ, IL-6, IL-10, IL-12 and IL-13 [156].

#### Effects of splenic cells on stroke

These responses in the spleen after CI may provide new opportunities for the development of novel stroke therapies. What effect does adoptive reinfusion of splenic cells have on stroke? Syngeneic transplantation of newborn splenocytes in a murine model of neonatal I/R achieved long-term survival of the grafts, exerted an influence on the microenvironment in the injured brain and showed improvement in behavioural tests 2 weeks after onset, but these parameters were not significantly different from those of the control groups after four weeks of brain injury [157]. The effect of adoptive transfer of splenic immune subsets on CI outcomes still depends on the splenic integrity of the mice [158]. From the above research, we can deduce that different subsets of splenic cells may play distinct roles in different pathological stages of stroke through the release of various cytokines.

### Brain-spleen cross-talk after stroke

The mechanism underlying the splenic responses after stroke and methods to improve the prognosis of stroke by intervening with the splenic reactions have become urgent issues in need of clarification. Although the exact mechanisms underlying the initiation of splenocyte responses to stroke have not been identified, several events, including ANS activation, release of CNS antigens and chemokine/chemokine receptor interactions, have been documented to be essential for efficient brain-spleen cross-talk after stroke.

#### ANS

Clinical and experimental studies have shown that the ANS is also involved in the regulation of immune-related pathological processes and neuroprotective effects after stroke [159, 160]. Symptoms of ANS dysfunction often occur after stroke, and in many cases, they show a special association with the location and exacerbation of the brain damage. Because many pathological risk factors of stroke are closely related to changes in ANS function, we can speculate that an interdependence exists between the ANS and stroke. The ANS may affect the immune system through many pathways. Many PIOs, including the spleen, are controlled by the ANS (mainly the SNS). At the same time, immune cells and organs also express various receptors for sympathetic neurotransmitters [159, 160]. SNS markers are increased and parasympathetic nervous system (PNS) markers are decreased after stroke [161]. Increasing SNS activity or decreasing PNS activity is associated with a worsening prognosis in patients with acute stroke [162–165]. Blocking SNS by chemical methods can effectively relieve the immunosuppression and the reduction in the splenic volume, which improve the outcomes of experimental animals after CI [166–168].

The CNS regulates immune system activity mainly through complex humoural and neural pathways, including the hypothalamic-pituitary-adrenal (HPA) axis, vagus nerve (VN) and SNS [169]. Elevated cortisone, corticosterone and metanephrine levels and associated lymphocytopaenia are often observed after extended brain infarction. The differential effects and complex interplay between the SNS and the HPA axis on systemic immune cells have to be considered when targeting the neurohormonal systems in the acute phase of severe stroke [170]. The hypothalamus is associated with the central function of the ANS by synchronising the neuroendocrine (glucocorticoid) response and cholinergic pathways, which together inhibit the release of inflammatory cytokines from peripheral T cells, monocytes and macrophages and promote the release of anti-inflammatory cytokines, such as IL-10. Similarly, norepinephrine (NE) released from dense neural networks throughout the brain and from peripheral organs, including the spleen, induces significant anti-inflammatory phenotypes in lymphocytes, monocytes and macrophages. In addition, the release of catecholamine from nerve endings can induce the release of acetylcholine (ACh) from splenic T memory cells, which can inhibit inflammation and increase the risk of infection after stroke [169]. A recent work showed that transcutaneous auricular VN stimulation (ta-VNS) reduced the infarct volume and induced angiogenesis in focal cerebral I/R rats. Ma suggested that the neurobehavioural recovery induced by ta-VNS might involve spleen-brain communication triggered by redistribution of growth differentiation factor (GDF)-11 [171].

**Fig. 5:**
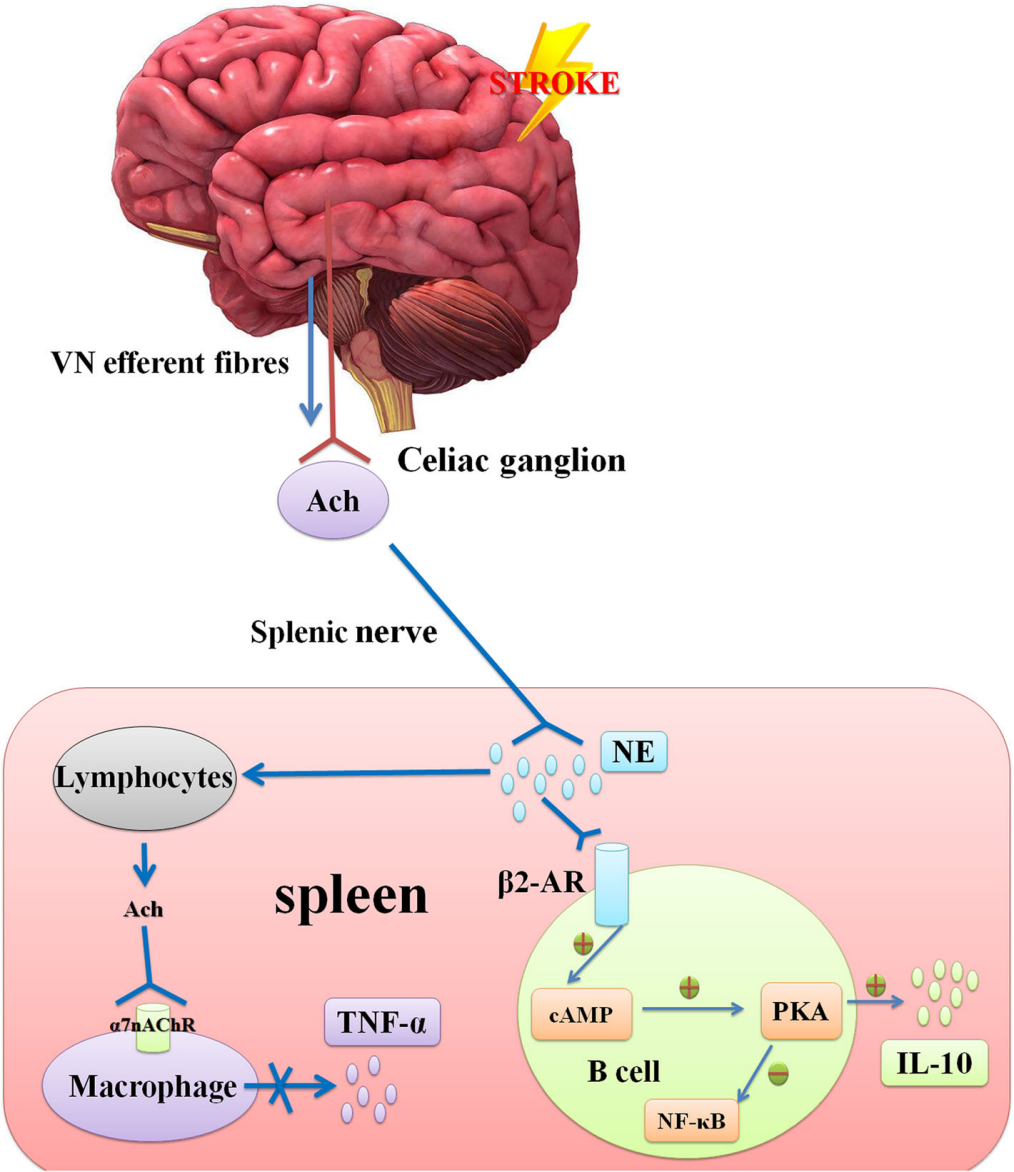
Splenic sympathetic nerve-mediated anti-inflammatory pathway after stroke. After the acute stage of stroke, “brain-spleen cross-talk” not only inhibits the splenic inflammatory response by activating the SNS (reducing the production of inflammatory factors, such as TNF-α) [172] but also induces IL-10 production by lymphocytes in the spleen through activation of the NE-mediated PKA/cAMP pathway in other inflammatory-related disease models [174, 175]

The brain and viscera interact through the ANS, and the VN, which contains 80% afferent fibres and 20% efferent fibres, plays multiple key roles in regulation of visceral homeostasis and anti-inflammatory processes [172]. These VN functions are mediated by many pathways, some of which are controversial. In the splenic sympathetic nerve-mediated anti-inflammatory pathway, VN fibres stimulate the splenic sympathetic nerve, causing NE release from the distal splenic nerve to act directly on the β2-AR of splenic lymphocytes, thereby inducing the release of ACh. Finally, ACh inhibits the release of TNF-α from spleen macrophages through the α-7-nicotinic ACh receptor (α7nAChR) [172]. At the same time, activation of the β2-AR receptor on some splenic lymphocytes may trigger activation of the cAMP-PKA pathway [173], which is related to inhibition of the NF-κB pathway and IL-10 production in the spleen [174, 175] (Fig. 5).

Generally, the immune response to stroke can be divided into two stages. The immune response at the early stage of acute stroke is pro-inflammatory and is driven by an increase in SNS activity. Later, immunosuppression starts when the spleen has depleted its immune cell reserve, and the risk of infection increases after stroke during this window [117]. Rasouli et al. also proposed a similar viewpoint of “brain-spleen inflammatory coupling” by which autonomic control of splenic macrophages could modulate systemic inflammation after injury. Stimulation of α/β-AR located on splenic macrophages leads to the release of TNF-α and IL-1β, which enhance and exacerbate inflammation. Conversely, parasympathetic stimulation of α7nAChR inhibits the release of these cytokines, thereby attenuating the inflammatory response to injury [176].

#### CNS antigens

In response to stroke, the ischaemic brain may secrete a variety of antigens that activate adaptive immune responses and recruit immune cells from the spleen. During acute stroke, neo-antigens, such as microtubule-associated protein 2 (MAP2), *N*-methyl-d-aspartic acid receptor subunit 2 (NR-2A), myelin basic protein (MBP) and myelin oligodendrocyte glycoprotein (MOG), can all be released into the periphery and captured by APCs, especially DCs and macrophages. This response is thought to eventually trigger the activation of T cell-dependent adaptive immune responses in the T cell zone [177, 178].

Although the immune system does not directly initiate the pathological process of stroke, stroke-induced immune activation has been increasingly recognised to enhance the neuropathological outcomes or nerve repair. Elucidating novel neo-antigens that are targets for immune cells offers unique insights into potential cellular and systemic consequences of autoimmunity during post-stroke neuronal plasticity. Mice with small infarct volumes exhibited high autoreactivity to MAP2 and MBP. This autoimmunity was maintained through splenic CD4^+^ and CD8^+^ T cells as well as CD19^+^ B cells during the first 10 days post-stroke [179]. An early splenic CD4^+^ T cell autoimmune response to neuronal and myelin antigens is associated with better recovery [170]. Ren et al. applied adoptive transfer of MOG-reactive splenocytes capable of secreting toxic Th1 cytokines (such as IFN-γ and TNF-α) to mice with severe combined immunodeficiency. This manoeuver resulted in an increased infarct size, increased neurological deficits and a higher accumulation of immune cells in the ischaemic brain in the treated mice relative to those of the control animals [180]. The MBP-specific phenotype of the splenocytes was obtained from donor animals 1 month after stroke and adoptively transferred to naïve recipient animals at the time of CI. Animals that received either MBP-specific TH1 or TH17 cells suffered worse neurological outcomes, and the degree of impairment correlated with the robustness of the MBP-specific TH1 and TH17 responses [181]. Hurn et al. put forward a hypothesis of “brain-spleen cell cycling” that stated that adaptive immune cells could be triggered through an encounter with CNS antigens in either soluble form or presented by macrophages or DCs [182].

#### Chemokines/chemokine receptors

Chemokines (CCL2, CCL3, CCL5, CX3CL1, CXCL8, CXCL12, etc.) are secreted by damaged central cells to recruit inflammatory cells into the damaged brain. The corresponding chemokine receptors are also increased in splenocytes after I/R [102].

CCL2 can effectively mediate monocyte/macrophage and neutrophil infiltration during CI [183]. Inadequate CCR2 expression results in decreased monocyte and neutrophil infiltration into the ischaemic brain, followed by decreased inflammation and cerebral infarction. Bao also showed that the CCL2-CCR2 interaction might play an important role in the distribution and migration of monocytes from the spleen to the injured brain [132]. Moxifloxacin treatment can effectively inhibit CCR2 expression in monocytes, thereby significantly reducing the infarct size after CI [183].

The CXCR4-CXCL12 axis is closely related to the pathology of ischaemic stroke. CI leads to a rapid and long-lasting increase in CXCL12 in the ischaemic penumbra. Transplanted GFP-labelled bone marrow cells are recruited in proximity to these CXCL12^+^ vessels and display characteristics of activated microglial cells. Therefore, we can speculate that CXCL12 plays an important role in homing of bone marrow-derived monocytes, which transform into microglia at the site of ischaemic injury [184]. The CXCR4 antagonist AMD3100 blocks the interaction between CXCR4 and CXCL12, which not only alleviates cerebral inflammation and cerebral infarction but also prevents splenic atrophy after tMCAO. Therefore, CXCR4-CXCL12 may play a regulatory role in the splenic response after stroke [185].

Many other cytokines have also been shown to play important roles in the recruitment of immune cells into the ischaemic brain. For example, CCL3 is closely related to the accumulation of monocytes and neutrophils in damaged brain tissue [186, 187]. CCL5 is involved in leukocyte infiltration after I/R [188]. CX3CR1-knockout mice show reduced neuroinflammation after focal CI, suggesting that CX3CL1 promotes post-stroke inflammation, which may be related to chemotaxis of monocytes, T cells and NKs [148, 189, 190]. CXCL8 has been considered as an important chemotactic factor for neutrophil recruitment after ischaemic stroke [191]. IP-10 expands the NK-induced damage of the BBB through CXCR3 [134]. The increased CXCL-1 and CXCL-2 levels in the brain tissue after stroke lead to accelerated leukocytes and particularly granulocyte accumulation and aggravate ischaemic tissue damage [192]. CCL20 is upregulated in the thymus and spleen 24 h after TBI and in the cortex and hippocampus 48 h after TBI [116]. The roles of these cytokines in splenic cell mobilisation warrant further study.

### Stem cell therapy targeting the spleen

**Fig. 6:**
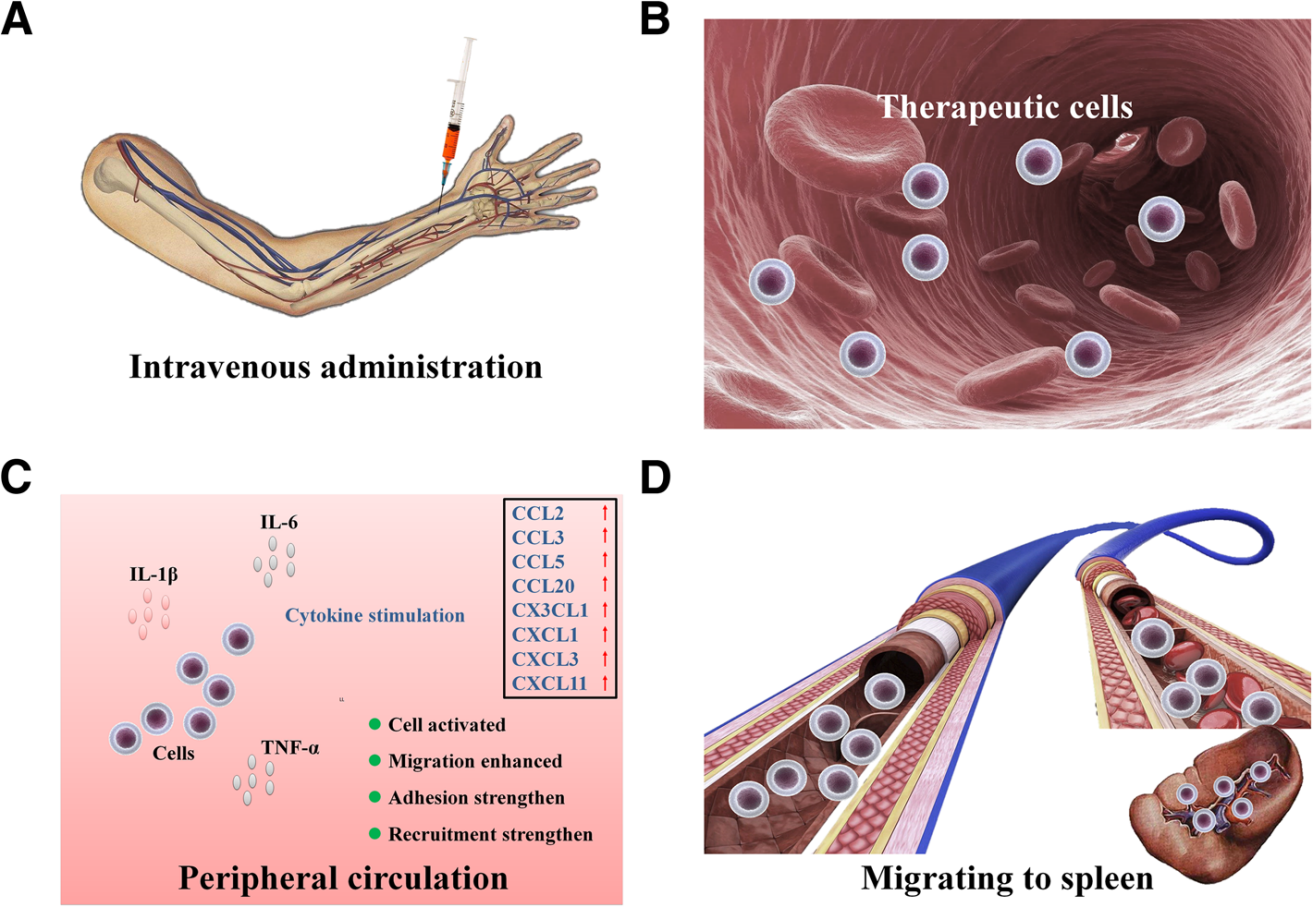
Therapeutic stem cells after stroke migrate to peripheral organs, such as the spleen. **a** After stroke, the patient was treated by intravenous injection of stem cells. **b**, **c** Stem cells in circulation are stimulated by IL-1β, IL-6 and TNF-α after stroke, and their chemokine ligand levels are elevated, thus enhancing the capability of stem cells to recruit inflammatory cells [194, 195]. **d** Through migration and adhesion, stem cells migrate rapidly to PIOs, such as the spleen, to play a regulatory role

In various experimental models, hUCBs [32–36], HSCs [35], BMSCs [36, 97, 193], hAECs [48] and NSCs [37] have been shown to reduce the neurological damage caused by stroke. Compared with that of intracerebral administration, all stem cells show better therapeutic effects when administered systemically. Stem cells migrate to the injured brain and spleen and in some cases have been shown to modulate the immune response to stroke [32, 35–37, 48], which may be one reason that this injection route is more efficacious. Ninety-five percent of BMSCs are found in the spleen following systemic administration after MCAO [36] (Fig. 6).

Intravenous hBMSCs preferentially migrate to the spleen and alleviate chronic inflammation in rats with CI. hBMSC treatment reduces the TNF-α level in the spleen after CI by 40%. Correlation analysis revealed negative correlations between hBMSC migration in the spleen and the infarct areas, peri-infarct areas, volume of MHCII^−^ activated cells in the striatum and TNF-α level in the spleen [31]. Even NSCs migrate to the spleen following ICH, but the therapeutic effect disappears after splenectomy. NSCs have been found to be in direct contact with CD11b^+^ cells in the spleen [37], which partly demonstrates that the neuroprotective effect of NSCs during stroke involves their interaction with the spleen.

**Fig. 7:**
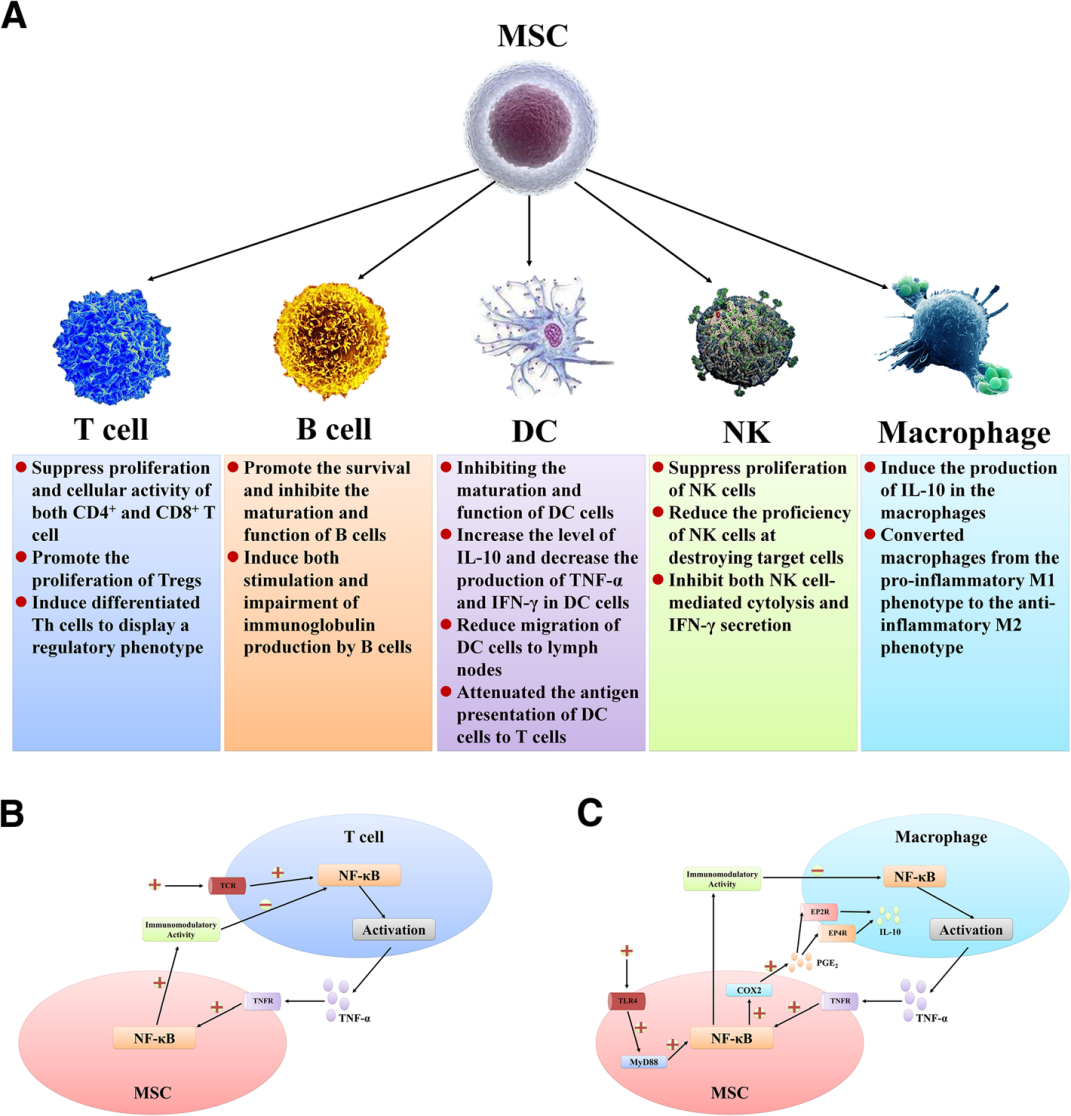
Effects of MSCs on various immune cells in circulation and in the spleen after stroke. **a** MSCs affect various immune cells in circulation and the spleen after stroke through soluble molecules and direct interactions [198–200]. **b** T cell activation triggers the expansion of T cell clones and secretion of TNF-α and other inflammatory factors. Then, TNF-α activates the NF-κB signalling pathway in MSCs located in inflammatory environments through the TNF receptor, thereby inducing MSC-mediated immunosuppression [194, 201, 202]. **c** Similar to T lymphocytes, TNF-α produced by activation of the NF-κB signalling pathway in macrophages also induces MSC-mediated immunosuppression, which inhibits macrophage activation and converts macrophages from the pro-inflammatory M1 phenotype to the anti-inflammatory M2 phenotype by blockade of NF-κB. During this process, NF-κB signal activation in MSCs upregulates COX2 expression, which is turn increases the synthesis of prostaglandin E2 (PGE2). The secreted PGE2 binds to EP2 and EP4 receptors on macrophages, thereby increasing IL-10 secretion by macrophages to reduce inflammation [203, 204]

hUCBs are another cell type that has been shown to affect the spleen during the pathological process of stroke. hUCBs alter cell populations in the peripheral circulation and spleen after pMCAO due to the interactions of all subpopulations together [196] (Fig. 7). Systemic administration of hUCBs 24 h after MCAO significantly alters splenic T cell responses to concanavalin A, decreases the proliferation activity of splenic T cells, decreases production of the inflammatory factors TNF-a and IFN-γ and increases production of the anti-inflammatory cytokine IL-10 [32, 196]. hUCBs also inhibit splenic atrophy in rats 48 h after MCAO. This effect is thought to be achieved by regulation of immune cells in the spleen by hUCBs after MCAO and inhibition of their release into the systemic circulation [24]. Kadam et al. used intravenous injection of CD34^+^-enriched hUCBs to treat CI mice; the experimental results showed that neurogenic niche proliferation and glial brain responses to CD34^+^-enriched hUCBs after neonatal stroke might involve interactions with the spleen and were sex-dependent [197].

hAECs are derived from the epithelial layer of the amnion, which is the sac that encloses the developing foetus and is attached to the placenta. Evans recently tested the efficacy of systemically delivered hAECs to improve a number of outcome measures in four animal models of ischaemic stroke [205]. Based on their experimental results, they put forward the hypothesis that administration during the acute phase (within 1.5 h) after ischaemic attack allowed the hAECs to migrate preferentially to the spleen and damaged brain; subsequently, cell apoptosis and inflammation were inhibited. Early brain infiltration of immune cells, aggravation of infarction and systemic immunosuppression were also alleviated [48].

HSCs injected intravenously 24 h after reperfusion were first detected at 24 h after injection in the spleen and later in the ischaemic brain parenchyma [35]. In addition, compared with that of the sham-operated control group, the immune environment after CI increased HSC migration to the spleen 72 h after reperfusion. In the absence of induction of an injury, the cells did not preferentially accumulate in the spleen [35]. HSC treatment reduced the infarct volumes, apoptotic neuronal cell death in the peri-infarct areas and immune cell (T cells and macrophages) infiltration into the ischaemic hemispheres. Moreover, HSC therapy decreased the TNF-α, IL-1β, CCR2 and CX3CR1 levels in the spleen after CI [35].

These experiments suggest that stem cell therapy works to some extent by regulating the immune response after stroke, especially at the spleen level, which may be crucial and is an important potential therapeutic target.

### Important therapeutic targets

Many factors and regulatory pathways change significantly during the entire pathophysiological process of stroke. They may play different roles in different pathological stages after CI. Fully understanding their effects on stroke may identify new targets for development of novel therapeutic strategies.

#### IL-10

IL-10 is a multicellular, multifunctional cytokine that regulates cell growth and differentiation and participates in inflammatory and immune responses. It is mainly produced by cells such as Tregs, Th2 cells and Bregs and currently is recognised as an anti-inflammatory and immunosuppressive factor. IL-10 is an important component of the endogenous repair mechanism after stroke. The IL-10 levels in the brain and spleen increase after stroke [90, 155, 156]. The use of exogenous IL-10 for anti-inflammatory therapeutic approaches has been shown to provide neuroprotection during ischaemic stroke [206]. However, an excessive IL-10 response can contribute to immunosuppression after CI, which worsens outcomes. Additionally, sex differences may exist in the role of IL-10 in stroke recovery [207].

**Fig. 8:**
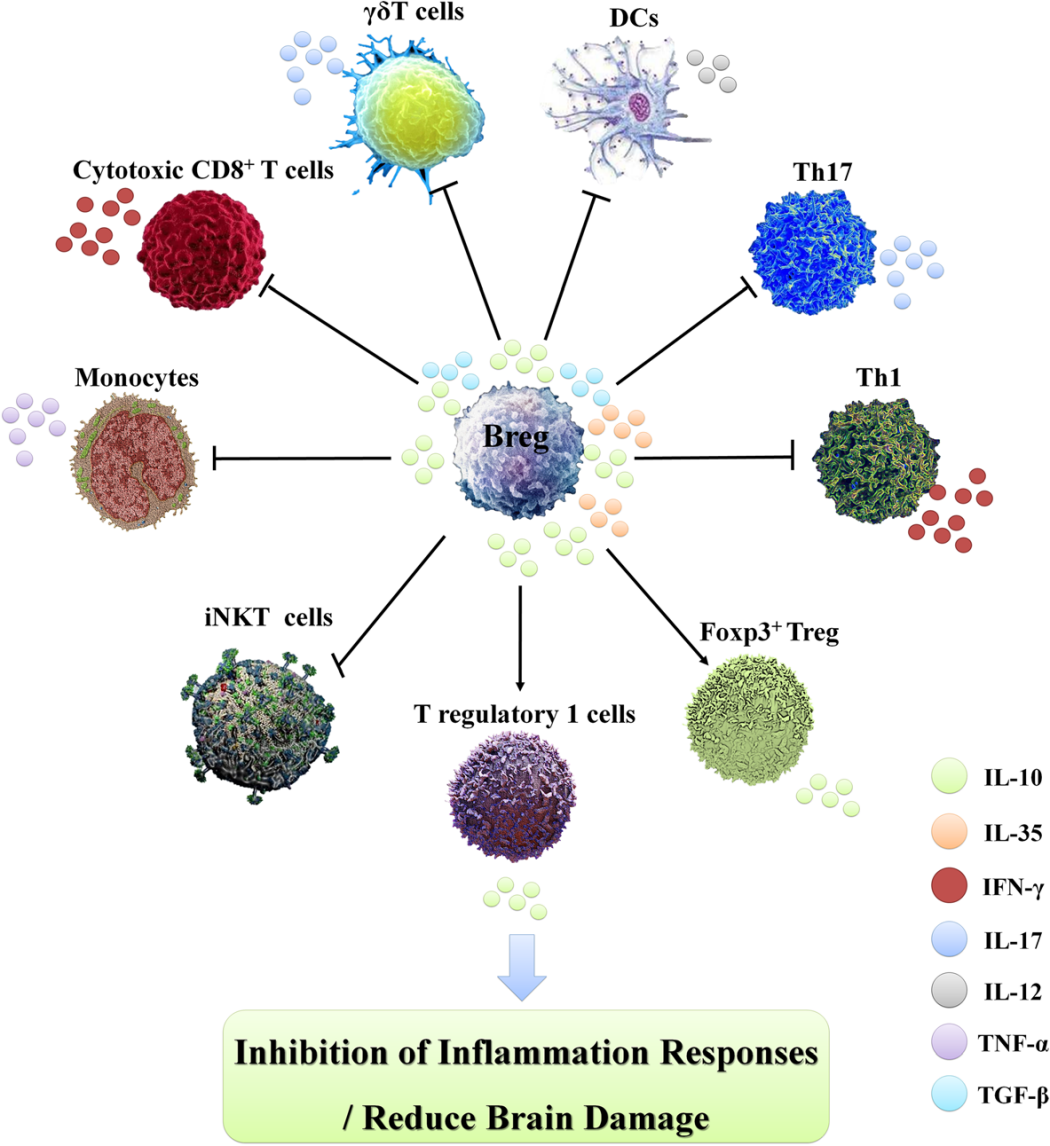
Effects of Breg cells on other immune cells after stroke. Through IL-10, IL-35 and TGF-β production, Bregs can inhibit the differentiation of pro-inflammatory lymphocytes, such as TNF-α-secreting monocytes, IL-12-secreting dendritic cells, Th17 cells, Th1 cells, IL-17^+^ γδT cells and cytotoxic CD8^+^ T cells. Bregs can also induce the differentiation of immunosuppressive T cells, such as Foxp3^+^ Tregs and T regulatory 1 (Tr1) cells and contribute to the maintenance of iNKTs. Therefore, Bregs result in immune regulation at sites of inflammation, such as the CNS

Adoptive transfer of IL-10-secreting cells is widely used for the treatment of experimental stroke. However, after the earlier use of Tregs [41], Bregs are attracting increasing attention. Adoptive reinfusion of spleen-derived Bregs can improve neurological injury and motor dysfunction after CI in experimental rats [34, 140]. Elevated Breg and Treg levels in the spleen at different time points after CI have been found in some studies investigating the preconditioning protection of I/R [208, 209]. In addition to their anti-inflammatory effects, Bregs can also promote the activation and proliferation of other anti-inflammatory immune cells and have a cascade amplification effects on the repair mechanism after CI [147, 210] (Fig. 8). Therefore, we have proposed that Bregs may represent an important potential strategy to rapidly start the endogenous protective mechanism during the early stage of stroke and increase the Breg levels in the body, especially in the spleen.

#### Interferon

IFN-γ is the only member of the type II IFNs and is produced only by activated T cells, NKs and natural killer T cells (NKTs). IFN-γ is a marker cytokine of Th1 cells that can activate APCs and promote the differentiation of Th1 cells by upregulating related transcription factors. Deletion of the IFN-γ gene has been shown to reduce brain damage after CI [137]. In the first 3 days after I/R, intraventricular administration of an IFN-γ neutralising antibody can protect the brain from CI injury [140]. IFN-γ participates in the Th1 inflammatory response by activating monocytes, microglia and macrophages. Since activation of microglia/macrophages is part of the cause of delayed cell injury following ischaemic injury, IFN-γ may play a role in the splenic response by aggravating the inflammation associated with ischaemic injury. Seifert et al. previously proposed that the spleen contributed to stroke-induced neurodegeneration through IFN-γ signalling [119]. IFN-γ is increased early in the spleen but later in the brain following I/R. Splenectomy reduces the IFN-γ level in the infarct after MCAO. The protective effect of splenectomy was eliminated by systemic recombinant IFN-γ accompanied by an increase in IFN-γ expression in the brain post-pMCAO [119]. Moreover, IP-10 has been shown to be a key factor in stroke-induced neurodegenerative diseases [154]. NKs participate in CI and promote neuronal necrosis through IFN-γ. IP-10 expands the damage of the BBB induced by NKs through CXCR3 [149]. Furthermore, hUCB therapy inhibits the proliferation of splenic T cells after MCAO by increasing IL-10 and IFN-γ production [32].

IFN-β is a type I IFN that binds to the IFN-α/β receptor. IFN-β exerts anti-inflammatory effects, and systemic administration of recombinant IFN-β has been used for the treatment of multiple sclerosis, which is a neuroinflammatory condition of the CNS [211]. Therefore, IFN-β has been considered to be able to decrease neuronal death and promote functional recovery after CI by limiting inflammation. In agreement with this view, IFN-β has been put forward as a candidate drug for the treatment of stroke. Several animal experiments have shown that systemic administration of recombinant IFN-β at different time points before and after MCAO results in neuroprotection [212–214]. This outcome may be related to endogenous IFN-β signalling not only to reduce CNS inflammation but also to reduce autoreactive T cell proliferation via inhibiting the antigen presenting capacity of astrocytes and microglia. Inácio’s research also showed that endogenous IFN-β signalling could alleviate local inflammation and regulate peripheral immune cells, thereby contributing positively to stroke outcomes [215].

#### Cholinergic anti-inflammatory pathway

As mentioned above, VNS causes prominent attenuation of the systemic inflammatory response evoked by CI in experimental animals. This effect is mediated by ACh stimulation of acetylcholine receptors on splenic macrophages. Therefore, the circuit is known as the “cholinergic anti-inflammatory pathway”, which casts the spleen as the major effector [216]. α7nAChR is considered to be an important target for alleviating the release of pro-inflammatory cytokines from macrophages and DCs.

Noradrenergic neurons provide innervation to all primary and secondary lymphoid tissues, including the bone marrow, spleen and lymph nodes [217, 218]. Most immune cells express one or more ARs, but β2-AR is the most widely distributed and mediates most of the effects of sympathetic nerves on immune function [219–222]. Sympathetic nerves affect the innate and adaptive immune responses through stimulating β2-AR. Most evidence shows that β2-AR activation has immunosuppressive effects on monocytes and macrophages [221, 222]. Stimulation of β2-AR-naïve CD4^+^ T cells (Th0) results in their differentiation into Th1 cells, which enhance cellular immunity, or Th2 cells, which decrease cellular immunity [221, 222]. Norepinephrine (NE) can also produce β2-mediated anti-inflammatory effects by releasing ACh from cholinergic spleen cells [221, 222]. Blocking adrenocortical receptors has been shown to inhibit the splenic response after pMCAO and reduce injury [123]. The spleen receives noradrenergic innervation from the postganglionic sympathetic neurons [223]. Splenic T cells are the source of ACh and may be a link between NE and splenic macrophage suppression [224]. β2-AR on T cells is essential for the anti-inflammatory effect of VNS [225, 226]. Splenectomy eliminates the role of VNS in increasing plasma NE, which supports the conclusion that plasma NE is released from the spleen into circulation during VNS [227]. The spleen plays a central role in the pathophysiology of the hyperinflammatory state triggered by threatening conditions, such as stroke, and splenic macrophages are the dominant source of pro-inflammatory cytokines [226].

Targeted therapy for α7nAChR on microglia and macrophages after stroke has long been considered an important potential strategy. α7nAChR expression on activated microglia and infiltrated macrophages after CI plays an important role in the pathological process of stroke. Nicotine therapy has been shown to significantly attenuate the increase in microglia and inflammatory cytokines (i.e., TNF-α and IL-1β) induced by CI through mediation of α7nAChR [228]. In addition, α7 agonists can reduce the infarct volume and functional deficits in different animal models of stroke [229, 230]. In contrast, blockade of α7nAChRs with a selective antagonist increases the infarct volume, which suggests some degree of α7nAChR stimulation by the endogenous agonist [230]. The endogenous choline released from damaged brain tissue may fulfil this role. The use of an allosteric modulator of α7nAChRs 6 h after tMCAO can reduce the infarct volume and improve neurological performance [231, 232]. In addition, regulation of α7nAChRs has also been shown to be associated with changes in M1 and M2 microglia/macrophages after CI [229].

### Multipotent adult progenitor cells

MAPCs are a unique type of adult adhesion cells (MSC-like) that extend understanding of how intravenous cell therapy participates in and regulates the peripheral immune system after stroke. MAPCs can be isolated from bone marrow and other tissues [233], are well characterised and can be easily distinguished from bone marrow mononuclear cells (BMMNCs) and MSCs based on their size [234], transcriptome [235], microRNA profile [236], differentiation capability [237] and secretome [238].

Intravenous injection of MAPCs has been shown to have beneficial effects on other CNS injuries, such as TBI [239]. MAPC therapy attenuates activated microglial/macrophage responses, preserves the BBB, reduces cerebral oedema and improves spatial learning after TBI [118, 239, 240], and MSPC treatment within 24 h after injury can achieve better outcomes [241]. After TBI, intravenous MAPCs also tend to migrate to the spleen [234].

MAPCs have become a new hotspot of cell therapy for stroke after BMSCs, hUCBs and HSCs. MAPCs can significantly inhibit the inflammatory reaction of injured brain tissue [242, 243] and improve the motor function and neurological outcomes of experimental stroke animals [242, 243]. Moreover, MAPCs exhibit more robust tissue sparing and mitigation of glial activation than MSCs regardless of whether intravenous or intraparenchymal administration is used [244, 245]. Similarly, recent animal data support a role for intravenous MAPCs in regulation of the peripheral immune system through specific interactions between MAPCs and splenocytes, thereby promoting stroke recovery and inhibiting splenic atrophy in the 24 h after CI. Similar to results obtained from testing hUCBs, stroke can also accelerate the migration of splenocyte MAPCs to the spleen and inhibit splenocyte apoptosis. In addition, MAPCs decreased the levels of CD3^+^, CD4^+^ and CD8^+^ cells in the spleen and increase Tregs after tMCAO compared with the levels in the vehicle-treated group [246]. In terms of inflammatory factors, IL-1β and TNF-α were significantly lower in the splenic cells of rats with I/R after MAPC treatment than those of the saline-treated animals, whereas the IL-10 level was higher [246].

MAPCs not only have a good protective effect on acute CI but also maintain a stable and sustained therapeutic effect on chronic stroke. Compared with those of the saline-treated group, the MAPC treatment group exhibited advantages in locomotor and neurological outcomes that persisted for more than 28 days. In addition, the results of a comparative trial of MAPCs for stroke in splenectomized and sham splenectomized mice also demonstrated that MAPCs could inhibit expansion of the infarct volume through a non-spleen-mediated mechanism, but the MAPC-induced IL-10 production after tMCAO was still dependent on an intact spleen. Moreover, no significant difference was found in the improvement of neurological outcomes between the splenectomy group treated with MAPCs 24 h after tMCAO and the splenectomy group treated with saline [246].

Preclinical animal data support the benefits of intravenous MAPC therapy for stroke. A phase I/II clinical trial is under way to test the safety and efficacy of MultiStem (the MAPC clinical-grade product) for treatment of patients with acute ischaemic stroke. Previous in vitro studies have shown that MACPs inhibit CD8^+^ T cells, which are harmful to stroke [247]. The MASTERS trial (MultiStem in Acute Stroke Treatment to Enhance Recovery Study) was conducted in 33 clinical centres in the USA and UK from October 2011 through December 2015. First, MultiStem treatment was proven to be safe. No infusion-related allergic reaction was observed in the MultiStem and placebo-treated groups, and no cases showed neurological worsening. MultiStem treatment can reduce the risk of life-threatening adverse events or death and secondary infections in stroke patients. Furthermore, MultiStem treatment also greatly shortened the time in the intensive care unit and the overall hospitalisation time compared with those of the placebo-treated patients. Compared with those of the placebo, MultiStem treatment significantly reduced the biomarkers of post-stroke inflammation (circulating CD3^+^ T cells and inflammatory factors). Importantly, MultiStem treatment significantly improved the chance for an excellent outcome at 1 year of onset [248, 250].

Currently, the phase III MASTERS-2 trial aims to expand our knowledge and understanding of treatment of ischaemic stroke patients with MultiStem and plans to start recruiting patients in 2018. Another MultiStem clinical study named TREASURE (Treatment evaluation of acute stroke for using in regenerative cell elements) was officially launched in 31 medical centres in Japan in 2017. The research project recruited 220 patients with acute ischaemic stroke, including speech or motor deficits, as defined by a National Institution of Health Stroke Scale (NIHSS) score of 8–20 at baseline. TREASURE is a randomised, double-blind, placebo-controlled, multicentre phase 2/3 trial to evaluate the efficacy and safety of intravenous administration of MultiStem® compared with those of a placebo in patients with ischaemic stroke [249].

## Discussion

Intravenous cell therapy can modulate the acute and adverse contributions of the peripheral immune system after ischaemic stroke [250]. A single intravenous administration of cells 24 to 36 h after stroke onset that can mitigate and rebalance the immune response to the initial focal ischaemic injury is sufficient for nerve repair and improvement of long-term outcomes [29].

As mentioned earlier, the activation, migration and participation of peripheral immune cells, and perhaps most importantly immune cells from the spleen, are critical steps in the pathophysiological progression after stroke. Therefore, we believe that targeted inhibition of peripheral blood immune cell (especially splenic cell) activation, reduction of inflammatory cytokine production and inhibition of their entry into the brain parenchyma through the BBB are key steps in attenuating the expansion of pro-inflammatory microglial activation, neuronal die-back and tissue loss.

**Fig. 9:**
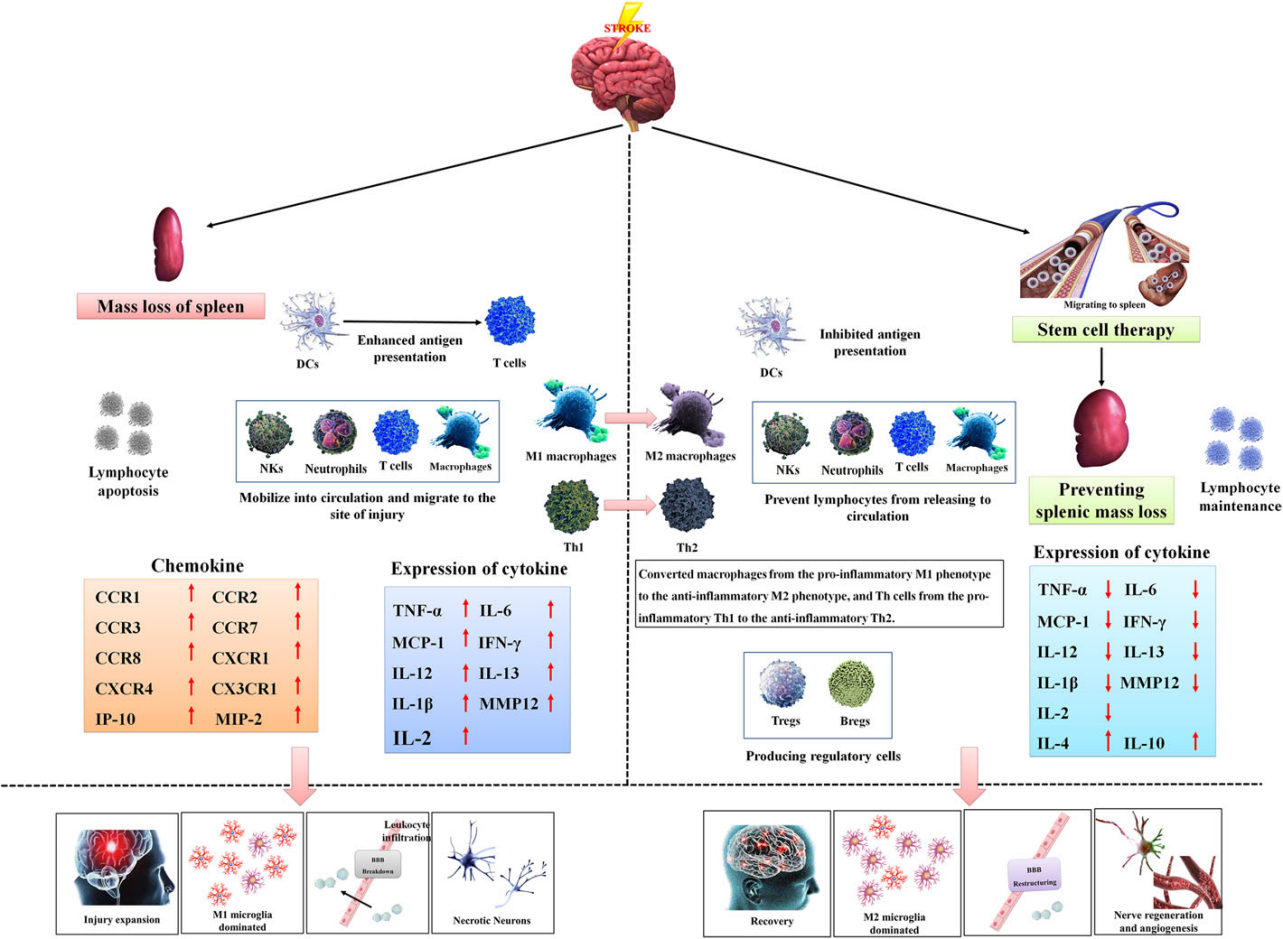
Stem cell therapy enhances recovery after stroke. In the untreated scenario, ischaemic stroke leads to activation of the peripheral immune system. During this process, the spleen atrophies, lymphocytes undergo apoptosis in the spleen, the inflammatory factor levels in the spleen increase, and inflammatory cells are released from the spleen into circulation. The antigen presentation of DCs is enhanced, and the levels of various chemokines are elevated. These pro-inflammatory mediators contribute to M1 microglia-mediated destruction of the BBB and CNS inflammation. Infiltration of leukocytes further aggravates inflammatory necrosis of neurons. Intravenous administration of stem cells reverses splenic atrophy and converts macrophages from the pro-inflammatory M1 phenotype to the anti-inflammatory M2 phenotype and Th cells from the pro-inflammatory Th1 phenotype to the anti-inflammatory Th2 phenotype. The inflammatory cytokine and cell levels in the spleen decrease, and anti-inflammatory cytokines and cells begin to be produced and released into circulation, which ultimately lead to less BBB restructuring and CNS inflammation and provide a favourable environment for nerve regeneration and angiogenesis

Simply inhibiting the participation of splenic components of the peripheral immune system in post-stroke pathophysiological processes allows tissue sparing but is not sufficient to enable neurological and locomotor benefits [246]. As mentioned above, splenectomy fails to provide long-term protection against ischaemic stroke, and delayed splenectomy neither reduces brain tissue loss nor alleviates sensorimotor and cognitive impairment [121]. These results suggest that the spleen may be a double-edged sword that plays completely opposite roles during different pathological stages of stroke. In the early stage of stroke, the spleen is mainly characterised by inflammation and harmfulness but then gradually transforms into an anti-inflammatory and protective phenotype. Therefore, inhibiting the inflammatory immune response of the spleen in the early stage of stroke and accelerating the initiation of its anti-inflammatory mechanism have become the keys for the use of stem cells in the treatment of acute stroke. We also summarise that many stem cells, including MAPCs, can simultaneously inhibit potentially harmful aspects of the innate immune system response to stroke while speeding up beneficial aspects or reparative responses, which confirms our viewpoint (Fig. 9).

Through a number of preclinical and clinical studies, we have obtained a certain understanding of the biological distribution of stem cells after injection for the treatment of stroke and their effects on many immune cell subsets and cytokines in the CNS and PIOs. However, little is known about the effects of stem cell therapy on the immune regulation mediated by the ANS and SNS. Many trials will provide an opportunity to better evaluate and examine hypothetical mechanisms, and we believe that stem cell therapy provides benefits for stroke; however, further preclinical and clinical studies are needed to advance a more comprehensive understanding. Evaluating the safety and efficacy of various stem cell therapies at multiple doses and at different time points may also yield new information.

## Conclusion

In summary, we can draw the following conclusions. First, the splenic response after stroke is critical for pathological damage and tissue repair. The key of immunomodulatory therapy for stroke may be to inhibit splenic inflammation at the early stage of pathology and accelerate the initiation of its anti-inflammatory/repair mechanism. Second, during the course of stem cell therapy for stroke, allowing more stem cells to migrate to the spleen may inhibit the splenic inflammatory response and accelerate the initiation of its anti-inflammatory/repair mechanism, which will have a better therapeutic effect than increasing the number of stem cells delivered to the injured brain tissue. Finally, inflammatory factors, such as TNF-α and IL-1β, produced after stroke can activate inflammatory pathways, such as NF-κB, in stem cells, thereby enabling stem cells to acquire stronger immune regulation potential. Therefore, stroke-like stimuli can be applied to stem cells for treatment before injection, which may lead to a better therapeutic effect. In the future, we believe that the spleen will become a potential target of various stem cell therapies for stroke represented by MAPC treatment. The research results will move closer to clinical application and ultimately benefit the majority of stroke patients.

## Abbreviations

Ach: Acetylcholine
ANS: Autonomic nervous system
APCs: Antigen-presenting cells
AR: Adrenergic receptor
BBB: Blood-brain barrier
BMMNCs: Bone marrow mononuclear cells
BMSCs: Bone marrow stem cells
Bregs: Regulatory B cells
CCL: C-C chemokine ligand
CI: Cerebral ischaemia
CNS: Central nervous system
COX: Cyclooxygenase
CXCL: C-X-C chemokine ligand
CXCR: C-X-C chemokine receptor
DAMPs: Damage-associated molecular patterns
DCs: Dendritic cells
GDF: Growth differentiation factor
hAECs: Human amnion epithelial cells
HPA: Hypothalamic-pituitary-adrenal
HPCs: Haematopoietic progenitor cells
HSCs: Haematopoietic stem cells
hUCBs: Human umbilical cord blood cells
I/R: Ischaemia/reperfusion
ICH: Intracerebral haemorrhage
IFN: Interferon
IGF: Insulin-like growth factor
IL: Interleukin
iNKTs: Invariable natural killer T cells
IP: IFN-induced protein
LPS: Lipopolysaccharide
MAP: Microtubule-associated protein
MAPCs: Multipotent adult progenitor cells
MBP: Myelin basic protein
MCP: Monocyte chemoattractant protein
MMP: Matrix metalloproteinase
MOG: Myelin oligodendrocyte glycoprotein
MSCs: Mesenchymal stem cells
MultiStem: The MAPC clinical product
NE: Norepinephrine
NGF: Nerve growth factor
NIHSS: National Institution of Health Stroke Scale
NKs: Natural killer cells
NKTs: Natural killer T cells
NLR: Nod-like receptor
NO: Nitric oxide
NPCs: Neural progenitor cells
NR-2A: N-methyl-d-aspartic acid receptor subunit 2
NSCs: Neural stem cells
p/t MCAO: Permanent/temporary middle cerebral artery occlusion
PAMPs: Pathogen-associated molecular patterns
PGE: Prostaglandin E
PIOs: Peripheral immune organs
PNS: Parasympathetic nervous system
PRR: Pattern-recognition receptors
ROS: Reactive oxygen species
SNS: Sympathetic nervous system
ta-VNS: Transcutaneous auricular VN stimulation
TBI: Traumatic brain injury
TGF: Transforming growth factor
Th: Helper T cell
TNF: Tumour necrosis factor
Tr1: T regulatory 1 cells
Tregs: Regulatory T cells
VEGF: Vascular endothelial growth factor
VN: Vagus nerve
α7nAChR: α-7-nicotinic ACh receptors
